# Early visual learning induces long-lasting connectivity changes during rest in the human brain

**DOI:** 10.1016/j.neuroimage.2013.03.050

**Published:** 2013-08-15

**Authors:** Maren Urner, Dietrich Samuel Schwarzkopf, Karl Friston, Geraint Rees

**Affiliations:** a UCL Institute of Cognitive Neuroscience, London WC1N 3AR, UK; b Wellcome Trust Centre for Neuroimaging, UCL Institute of Neurology, London WC1N 3BG, UK

**Keywords:** Resting state connectivity, Hippocampus, Striatum, Early learning, fMRI, Stochastic DCM

## Abstract

Spontaneous fluctuations in resting state activity can change in response to experience-dependent plasticity and learning. Visual learning is fast and can be elicited in an MRI scanner. Here, we showed that a random dot motion coherence task can be learned within one training session. While the task activated primarily visual and parietal brain areas, learning related changes in neural activity were observed in the hippocampus. Crucially, even this rapid learning affected resting state dynamics both immediately after the learning and 24 h later. Specifically, the hippocampus changed its coupling with the striatum, in a way that was best explained as a consolidation of early learning related changes. Our findings suggest that long-lasting changes in neuronal coupling are accompanied by changes in resting state activity.

## Highlights

• Early learning of sensory task changes hippocampal activity.
• Coupling changes between hippocampus and striatum.
• Resting-state changes are consolidated during sleep.
• Stochastic DCM is a tool for resting state analysis.

## Introduction

Until recently, functional MRI (fMRI) studies have focused on how brain activity changes with task performance or sensory stimulation. However, even at rest – in the absence of a task or stimulation – fMRI signals show spontaneous fluctuations that exhibit spatiotemporal correlations in networks of functionally connected areas (Biswal et al., 1995; Fox and Raichle, 2007; Raichle, 2010). These networks continue to covary during sleep (Fukunaga et al., 2006) and under anesthesia (Vincent et al., 2007). They show high consistency and reproducibility across subjects and sessions over the short-term and long-term, using different variations of independent component analysis (ICA) (Damoiseaux et al., 2006) and group ICA (Zuo et al., 2010). The reproducibility in healthy young individuals compares to that of activations elicited by motor paradigms (Meindl et al., 2010). Furthermore, there is a close correspondence between the activation networks – of almost 30,000 human participants of fMRI studies – with resting state networks (Smith et al., 2009). The interplay between spontaneous and evoked activity has been of particular interest. For example, in the visual cortex, spontaneous fluctuations determine the variability in cortical responses and perception associated with presentation of a simple visual stimulus (Schölvinck et al., 2012).

The effect of spontaneous fluctuations on evoked responses associated with perception raises the complementary question of whether systematic changes in evoked responses, for example present during learning, might subsequently alter spontaneous fluctuation. The mechanism we have in mind here is that experience dependent (associative) plasticity may change synaptic connections and ensuing neuronal activity in the local circuits affected. As the implicit short term and immediate long-term potentiation is consolidated the associated changes in spontaneous neuronal activity should persist and be measurable in terms of changes in effective connectivity. A growing number of studies have investigated this adaptive modulation of resting state networks. Changes in spontaneous fluctuations have been shown after visuo-motor learning (Albert et al., 2009), episodic memory tasks (Tambini et al., 2010), and language tasks (Hasson et al., 2009).

Visual learning is one way in which systematic changes in cortical responses and perception can be induced. Intensive training on a simple shape identification task over several days can change resting state functional connectivity between visual and fronto-parietal cortices (Lewis et al., 2009). This indicates that visual learning can have lasting effects on spontaneous brain activity through experience dependent plasticity. But such effects occur only after several days of training. The early phase of visual learning occurs much more rapidly—and is often ignored in typical visual learning experiments. However, learning entails a rapid consolidation process that starts within a single training session (Seitz et al., 2005) and that occurs in any experiment, independent of modality. The specific changes in spontaneous activity in task-responsive brain areas in response to this early learning (that occurs in any experiment, independent of modality) perhaps more typical of real-world environments (Brovelli et al., 2008; Shtyrov, 2012) remain unknown. With regard to visual learning, both sensory and non-sensory areas (Adab and Vogels, 2011; Goldstone, 1998; Seitz and Watanabe, 2005; Shibata et al., 2011), appear to be involved. Outside the sensory cortex, single-neuron and functional MRI studies have implicated the lateral intraparietal area (Law and Gold, 2008), lateral parietal cortex (Kahnt et al., 2011), subcortical structures like the hippocampus (Graham et al., 2006; Lee et al., 2005) and the caudate nucleus (Ding and Gold, 2010). Recently, sub-areas of the medial temporal lobe (MTL) including the parahippocampal cortex and subiculum have been implicated in rapid and incidental statistical learning in a visual paradigm (Schapiro et al., 2012). While MTL regions and, importantly, the hippocampus – including its connections to the striatum – have been traditionally linked to memory processes such as memory consolidation (Battaglia et al., 2011), their role in perceptual learning has only been examined more recently (Buckley, 2005).

Memory consolidation refers to the processes underlying the stabilization of memory traces acquired during initial encoding (Dudai, 2012); where the importance of sleep for consolidation is well-established (Wang et al., 2011). Previous studies of changes in resting state activity in response to recent experiences (Albert et al., 2009; Lewis et al., 2009; Tambini et al., 2010) have not examined long-term changes in spontaneous fluctuations in the resting state. This is probably due to the fact that it requires a more extensive study design. However, we were particularly interested in these potential long-term changes as markers of experience dependent plasticity induced by the early learning phase.

Therefore, we used a paradigm with only one relatively short learning session that promoted rapid perceptual learning. We hypothesized that rapid perceptual learning would be accompanied by changes in spontaneous activity in brain structures whose responses changed during learning. Furthermore, we predicted that resting state changes would persist following consolidation. We tested this hypothesis by acquiring resting state time-series using functional MRI before and after a standard perceptual learning experiment. During the experiment participants learned to discriminate a motion stimulus. We measured brain responses during task learning to identify regions whose responses were correlated with the learning in each individual. Crucially, we also acquired independent measures of resting state brain activity before and immediately after learning. The following day, we repeated the paradigm without the learning. We used stochastic dynamic causal modeling (DCM) to evaluate resting state effective connectivity (Li et al., 2011) between regions identified in the learning session. Specifically, we tested for learning dependent changes in effective connectivity (during rest) immediately after the learning session and after consolidation of these putative changes on the following day.

## Materials and methods

### Participants

16 right-handed healthy volunteers (7 female, 19–33 years of age, mean age 25.4 years) with normal or corrected to normal vision gave written informed consent to participate in the study consisting of two scanning sessions at two consecutive days. 11 of the 16 participants learned the motion coherence task and were included in the data analysis (3 performed at ceiling level and were excluded because we did not expect to see any neural changes in the absence of behavioral improvement; 2 were not able to learn the task as disclosed by their persistently low performance). The study was approved by the local ethics committee.

### Stimuli and task design

A random dot motion coherence stimulus was used. The level of dot motion coherence was set to 20%, which is close to the perceptual threshold and has been successfully used for naïve participants previously (Vaina et al., 1998). Further stimulus parameters were chosen according to the results of a behavioral piloting study of 15 participants. All participants performed 25 task and 25 control blocks, each consisting of 16 trials. A presentation time of 0.3 s was used in 7 subjects and 0.4 s in the remainder. The longer presentation time resulted in ceiling performance for 6 of the 8 participants. Therefore, we chose a 0.3 s presentation time for the scanning paradigm. The following stimulus parameters were used: dot speed: 10°/s, dot life time: 6 frames, response time: 1.5 s, number of dots: 200. White dots were presented in a central circular aperture covering a 3.14° visual angle on a black background. Participants were asked to focus on a white fixation square at the center of the screen throughout the experiment and no feedback was given.

During trials of the motion task, 80% of the dots were moving in random directions across the screen, while 20% of the dots were moving coherently to the left or right. The coherent direction was chosen randomly. Participants used their right hand and a keypad to report the direction of motion; i.e. left or right after the stimulus had disappeared. During control trials the dots were static and a little arrow, pointing either to the left or right, replaced the central fixation square. In these trials participants reported the direction of the arrow.

In total, each participant completed 800 trials—400 trials of the motion learning task and 400 trials of the control task, divided into 25 blocks of 16 trials each. The 25 blocks were spread over 5 runs, i.e. the scanner was restarted after 5 blocks—allowing participants to rest between runs. Each block of the motion task was followed by a block of the control task or vice versa.

### Experimental procedure

**Fig. 1:**
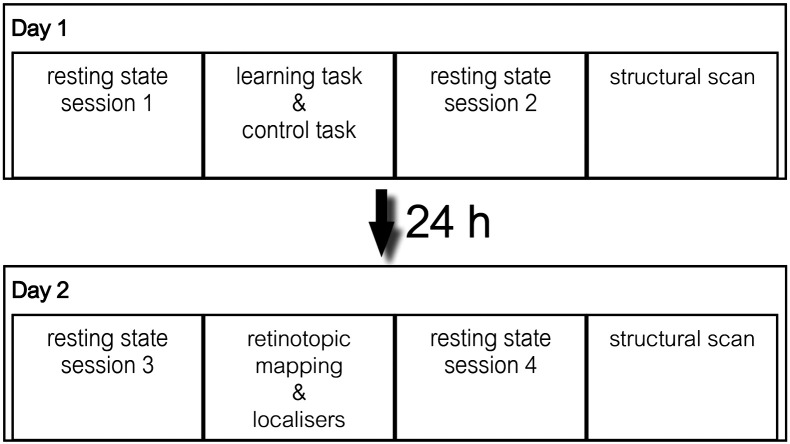
Experimental paradigm. Participants were scanned on two consecutive days for about 90 min each day. Two resting state runs were acquired each day, preceding and following the learning task or a retinotopic mapping respectively. A structural scan was acquired on both days.

To address potential changes in resting state connectivity due to learning and consolidation, participants were scanned on two consecutive days and brain signals were measured in four resting state runs: one before task performance, one directly after task performance, and two at the second day. These were repeated at the same times as the rest runs on the first day. Participants underwent standard retinotopic mapping and a V5/MT localizer in the scanner between the two rest runs of the second day (see Fig. 1).

Before entering the scanner on day one, participants were familiarized with the task, but did not pre-train (to ensure perceptual learning during scanning). Task instructions emphasized that accuracy was more important than speed when responding. Both scanning sessions lasted about 90 min (see Fig. 1 for details), and were separated by 24 h for each participant. During resting state runs participants were asked to close their eyes, relax, and to not fall asleep. The order of motion and control conditions in the learning task was counterbalanced over subjects.

### Behavioral analysis

Behavioral data were analyzed using inverse efficiency (IE)—a simple measure that combines reaction time and accuracy; where IE = mean reaction time / accuracy (Graham et al., 2006). Single trial reaction times that deviated from the mean of the respective block by more than three standard deviations were excluded. IE was calculated for each block (n = 25) and raw values were fitted to an exponential function of the form *y* = *ae*^− *bx*^ where *a* represents the amplitude and *b* the learning rate. The ensuing estimates of inverse efficiency were used as a parametric modulator of the stimulus regressors in the first level (within-subject) analysis of the functional data acquired during the learning task (see below). These regressors modeled learning related adaptation of BOLD responses.

### fMRI data acquisition

A 3 T Trio MRI Scanner (Siemens Medical Systems, Erlangen, Germany) with a 32 channel head coil was used to acquire functional data with a standard echo planar imaging (EPI) sequence (matrix size 64 × 64; field of view 192 × 192 mm; in plane resolution 3 × 3 mm; 32 slices in ascending acquisition order; echo time 30 ms; acquisition time per slice 68 ms; TR 2.176 s). Each run of the learning task comprised 246 volumes, and each resting state acquisition comprised 276 volumes. On both scanning days, B0 field maps were acquired to correct for geometric distortions in the EPI images. Also a structural T1-weighted scan was acquired on both days (matrix size 256 × 240; field of view 256 × 240 mm; in-plane resolution 1 mm × 1 mm; 176 sagittal slices of thickness 1 mm; echo time 2.48 ms; acquisition time per slice 7.92 ms). During scanning, respiration volume and cardiac pulse were measured using a breathing belt placed around the participants' waist and an MRI compatible pulse oximeter attached to one of the fingers. These data, together with scanner slice synchronization pulses, were sampled using Spike2 (Cambridge Electronic Design Limited, Cambridge, UK) and used for physiological noise correction.

### fMRI data analysis

Functional data were analyzed using SPM8 (http://www.fil.ion.ucl.ac.uk/spm/software/spm8/) and DCM12 was used for dynamic causal modeling of effective connectivity. To allow for T1 equilibration, the first five images of each run were discarded. Pre-processing of the data involved mean bias correction, realignment of each volume to the first volume of each run, coregistration of the functional data to the structural data of each day, coregistration of the structural scan (and functional volumes) of the first day to that of the second day, normalization to the MNI template brain and smoothing by an 8 mm Gaussian kernel. The task data were filtered with a standard 128-s cut-off and the resting state data were filtered with a 256-s cut-off, high-pass filter to remove low-frequency drifts—including differences between runs, while preserving as many of the spontaneous fMRI fluctuations as possible (Birn et al., 2007). Physiological data (respiration and heart beat) were modeled using an in-house developed MATLAB toolbox (Hutton et al., 2011) based on RETROICOR (Glover et al., 2000). This resulted in a total of 17 regressors. The resulting regressors were included as confounds in the first level analysis for each participant. Movement parameters were also included as confounds. No global signal regression was performed.

#### Perceptual learning session

Regressors modeling the stimuli were formed by convolving boxcar functions encoding each condition with a canonical hemodynamic response function—where stimulus functions modeling learning blocks were parametrically modulated by the fitted values of inverse efficiency (IE). These stimulus functions model perceptual learning related changes in responses evoked during the learning task. Contrasts of first level parameter estimates were used to perform a random effects analysis over participants in the usual way. This involved estimating (contrasts of) parameters encoding the effects of interest using a standard linear convolution model at the first (within-subject) level (over all five task runs) and then passing the resulting contrast images to one sample *t*-tests at the second (between-subject) level. The resulting statistical parametric maps (SPMs) were used to test for differences between the learning and the control task, the learning task and the fixation baseline, and the effects of learning (i.e. testing for a parametric modulation of the learning task effects). The anatomy toolbox (Eickhoff et al., 2005) was used to anatomically designate activated areas.

#### Psychophysiological interaction analysis

The peak activation – elicited by the effect of perceptual learning – was used as region of interest (ROI) for the analysis of the resting state data. Time series of this ROI were extracted for all four resting state runs and included as regressors in a first level general linear convolution model, together with the nuisance regressors. Again, resulting contrast images were passed to one sample *t*-tests at the second (between-subject) level and the resulting SPMs were used to test for changes in the coupling with the region defined during the learning task. More precisely, the four rest runs constituted two main effects, i.e. the main effect of day (rest 1 and 2 vs. rest 3 and 4) and the main effect of time (rest 1 and 3 vs. rest 2 and 4). The interaction of the two effects, i.e. day × time, was used to test for changes in the coupling between the learning related ROI and any other brain region (regression slope of regional activity on the activity of the ROI). Participant-specific peak coordinates of the learning related region were used. The peaks (p < 0.05, uncorrected) were within 16 mm of the second-level (between subject) peak and within the specific anatomical region, as defined by the SPM Anatomy toolbox (Eickhoff et al., 2005). Together with the learning related region, the region showing the most significant psychophysiological interaction (over subjects) was used for subsequent dynamic causal modeling of changes in their effective connectivity.

#### Dynamic causal modeling

DCM models neuronal dynamics in terms of directed and reciprocal influences among brain regions. Stochastic DCM allows one to model spontaneous or endogenous (non-controlled) activity. It does not require any input usually associated with experimental manipulation. Two subject-specific ROIs defined by the learning task and the psychophysiological interaction analysis were used as the nodes for 10 different models of changes in extrinsic connectivity. Regional activity in each ROI was summarized with its principal eigenvariate, adjusted for nuisance variables, based on voxels within 8 mm of subject-specific peaks. All four runs were concatenated into a single time series and parametric modulators were used to model learning-related changes in effective connectivity, plus potential consolidation of these changes.

More precisely, run-specific differences – in terms of the (bilinear) modulation of the average connectivity over all four rest runs – were modeled with three different parametric modulators. First, we modeled non-specific adaptation (i.e. the effect of “run”) due to time in the scanner by weighting the four different rest runs accordingly by [0 1 0 1]. Second, we added the effects of visual learning – following the learning phase – using the following weights [0 1 0 0]. Finally, a consolidation model comprised adaptation effects, i.e. [0 1 0 1], and learning effects that persisted during the second day with the following weights [0 2 1 1]. Crucially, the learning and consolidation models have two bilinear coupling parameters per connection that control the relative expression of adaptation and learning (or consolidation) respectively. We applied the models of coupling changes, – including a null model with no changes in coupling – to different permutations of connections: forward connections from one region to another, backward connections from one region to another, and bilateral connections, involving both forward and backward connections. This resulted in models with the same extrinsic reciprocal connections between two nodes, but different modulations of those connections. All models were fitted to the concatenated time series of the rest runs using generalized (Bayesian) filtering (Li et al., 2011). To evaluate the relative evidence for each of the 10 models, we compared the (variational free energy approximation to) log evidence. We used Bayesian Model Selection to select the model with the greatest evidence given the data. More precisely, we used relative log evidences, i.e. the model with the least evidence was subtracted from each model. This fixed effects model comparison was used because we assumed that the same model accounted for the data generated by every participant. A difference of three between log evidences – which corresponds to a relative evidence (Bayes factor) of about 20:1 – was used as the criterion for model selection.

For quantitative interpretation, the changes in effective connectivity under the winning DCM were computed by multiplying the appropriate bilinear parameters with the run-specific weights as specified above. Thus, for each participant each connection between the two regions included in the model was described by four values, reporting the connection strength in each resting run, relative to the first.

Non-specific adaptation between the first and second scanning day was not modeled, because we assumed that resting state connectivity would not show cumulative changes over successive days when learning had only occurred on the first day. Furthermore, we emphasize that the consolidation model did not simply represent a non-specific change in effective connectivity on the second day: it had to change in proportion to the learning-dependent changes on the first day.

## Results

### Participants showed early rapid learning of the motion task

**Fig. 2:**
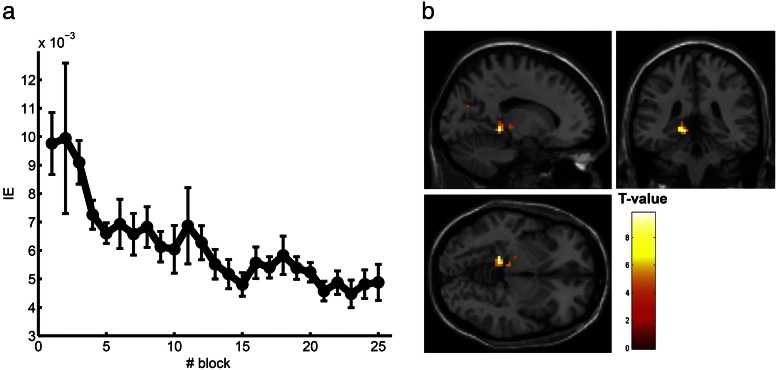
Behavioral learning and hippocampal activation. a) Participants learned the motion task. Inverse efficiency (IE) is plotted for every block of the task (n = 25). Data are averaged over all participants (n = 11) who learned the task successfully. Error bars show SEM. b) Learning activated the hippocampus. The fitted inverse efficiency values of the learning task were used for the plotted contrast. Statistics were significant at p < 0.05, FWE corrected. Images show activation at p < 0.001 (uncorrected).

Participants completed 400 trials of the motion task and 400 trials of the control task. Performance was measured using inverse efficiency (IE). The IE values of each block were fitted with an exponential function. See Behavioral analysis for details. See Fig. 2a for an overview of the learning. The fitted IE values entered the analysis of the functional neuroimaging data as a parametric modulation of the stimulus regressors in the first level (within-subject) analysis of the learning run. All participants who learned the task performed (as expected) at ceiling on the control task throughout the 25 blocks (mean of all participants over all blocks: 99% correct, range between participants: 97% to 100% correct).

### Motion task activated visual, frontal and parietal areas

After pre-processing, we first identified regions showing activity specific to the motion task by contrasting the blocks when participants performed the motion task with the fixation baseline. We found a bilateral network of visual areas, including V5/MT, as well as inferior parietal and orbitofrontal cortex (all p < 0.05, FWE corrected). See Table 1 for an overview. Next, we examined activations associated with the motion task compared to the static control task and found these in the inferior parietal cortex and the right insula cortex (all p < 0.05, FWE corrected), as well as in the visual cortex extending into V5/MT and medial temporal regions, and in the medial frontal cortex (all p < 0.001, uncorrected). See Table 2 for an overview.

### Early learning-related modulation of hippocampal activity during task performance

Using the IE-based parametric regressor, which modeled participant-specific learning on the motion task, we tested for regions whose responses adapted with performance. This analysis identified the left hippocampus (left subiculum, MNI coordinates (x = − 15, y = − 37, z = − 5), t = 9.77, p = 0.04, FWE corrected) (see Fig. 2b). The anatomy toolbox assigned the activation to the left subiculum with a 100% probability. None of the motion-activated areas given in Tables 1 and 2 showed any learning related changes (p < 0.001, uncorrected).

### Learning-related changes in connectivity during rest

Having identified the hippocampus as the key region whose activity changed significantly with perceptual learning (as indexed by participant-specific changes in performance) we next explored how the resting state connectivity of this region changed after learning. We first identified candidate regions whose connectivity with the hippocampus changed between resting state runs using a psychophysiological interaction analysis (Friston et al., 1997). These regions were then used in a dynamic causal model to examine changes in effective connectivity with the hippocampus. Using the independently acquired resting state data, we extracted the time series of the participant-specific hippocampal peak voxels for all four resting state runs and tested for changes in the coupling of the hippocampal region of interest with learning using PPIs.

To test for these changes, we treated the resting state runs as a 2 × 2 factorial design. Testing for the interaction between the two main effects of “run” (i.e. run one and three vs. run two and four) and “day” (i.e. run one and two vs. run three and four) we found that bilateral striatal loci showed changes in coupling with the hippocampus between runs that were significantly greater on the first compared to the second day (MNI coordinates (x = − 21, y = 8, z = 2), t = 3.59, p = 0.002, uncorrected; MNI coordinates (x = 21, y = 14, z = 4), t = 4.94, p < 0.001, uncorrected). No other regions showed a run by day interaction (p < 0.001, uncorrected) and we used the striatal region for the dynamic causal modeling.

### Dynamic causal modeling

Our subsequent tests for learning-related changes in effective connectivity (i.e. plasticity), and potential consolidation of these changes, were based on Bayesian model comparison using stochastic DCM (Li et al., 2011). Our models differed in terms of when and where changes in connectivity were expressed, i.e. specifically characterizing the forward and backward connections between the left hippocampal and striatal regions identified by the conventional SPM and PPI analyses. Our hypotheses were not about the existence of connections, but whether there were changes in specific connections between these areas across the different rest runs. Therefore, we considered four types of models: first a null model without any changes in connectivity (*null*). Second, we considered non-specific adaptation (*adaptation*), i.e. changes due to the main effect of “run”. Third, a learning-specific change expressed on and only on the first day at run two was added to the adaptation effect (*learning*). Finally, a consolidation model (*consolidation*), in which learning-specific changes on the first day did not disappear but were consolidated – at half their level – by the second day was added to the adaptation effect. Practically, each of these four models was specified with modulatory (bilinear) effects mediated by run-specific inputs that had different between run values but were fixed over the duration of each run. These four profiles of coupling changes between runs were applied to different permutations of connections; namely, either forward or backward or both forward and backward between hippocampus and striatum. This produced ten unique models, because the three null models for different architectures were identical. This model comparison is quite subtle, in the sense that we tested for the presence or absence of changes in the context of full connectivity—not the presence or absence of connections *per se*.

**Fig. 3:**
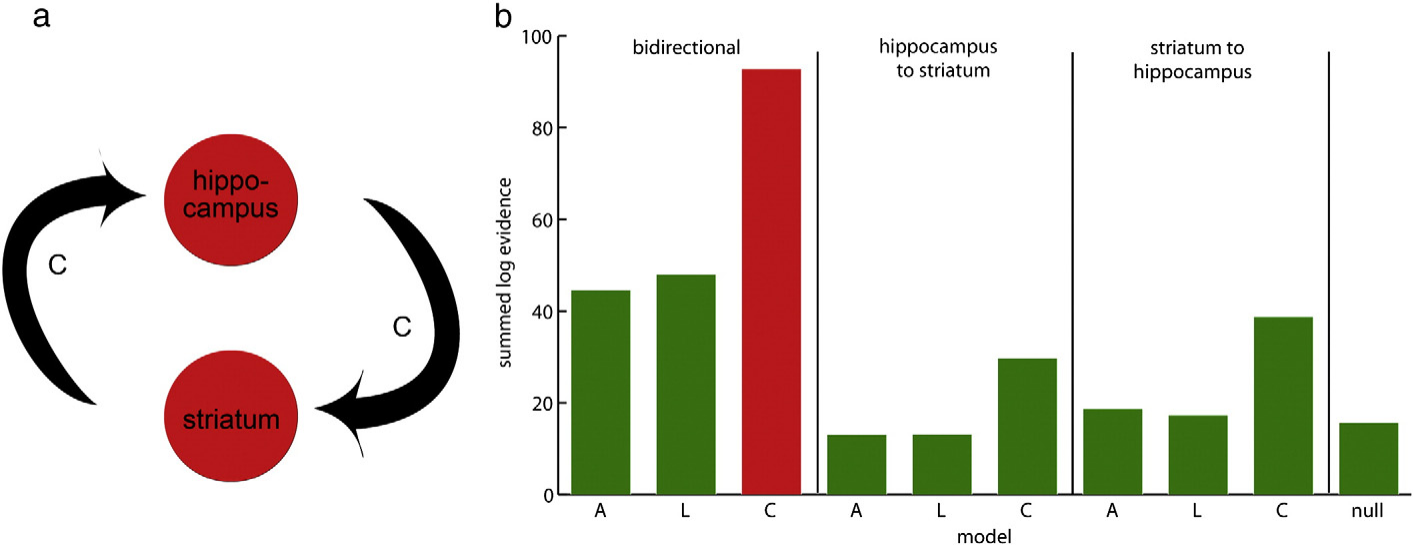
Winning model and summed (group) log evidence for all models. a) Schematic description of the model with a bidirectional connection between the hippocampus and the striatal region. The graphic shows which connections were modified by a consolidation pattern (see Results for detailed explanation). b) The model plotted in a) showed the highest evidence (marked in red). Plotted is the summed log evidence per model relative to the model with the least evidence. The winning model was the same for almost all participants (10 out of 11). A = adaptation, L = learning, C = consolidation, null = no modification.

**Fig. 4:**
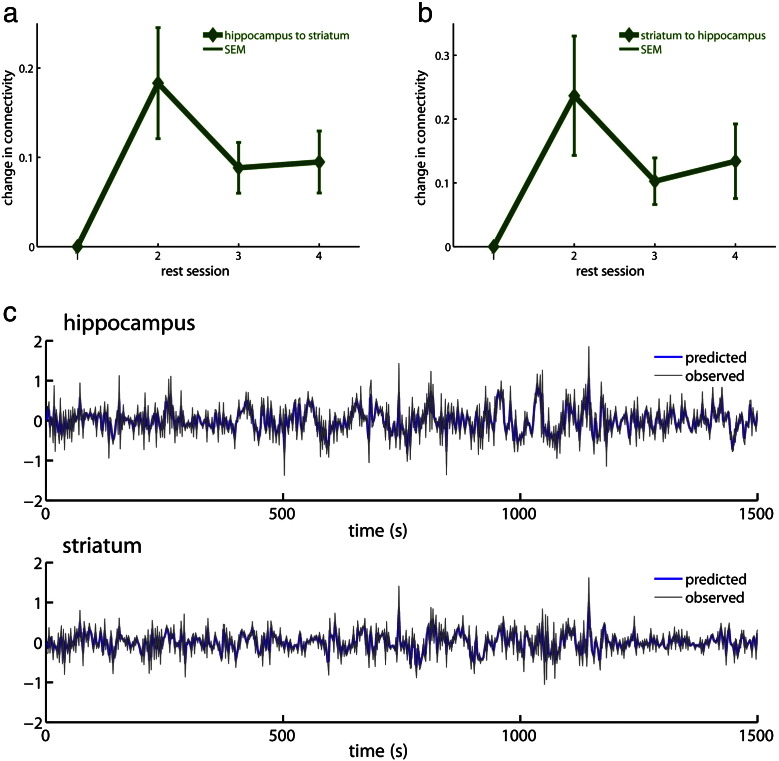
Parameter estimates and model fitting reflected consolidation. Parameter estimates for the modulation of the intrinsic connection from a) hippocampus to striatum and from b) striatum to hippocampus. After a big increase directly after the learning in rest run 2, the change in connectivity was preserved at a lower level on the second scanning day for both rest runs (i.e. rest runs 3 and 4). Plotted are the average values for all participants who learned the task (n = 11), error bars indicate the standard error of the mean (SEM). c) Overlay of observed (gray) BOLD time-series during rest with the time-series as predicted by DCM (blue). The two regions included in all tested models are shown for a representative participant.

Fulfilling our predictions of higher evidence for the learning or consolidation models, we found the highest log evidence for the consolidation model with a bidirectional change in connection strength between the hippocampus and striatum (see Fig. 3a for an illustration). Remarkably, this was the winning model for 10 out of 11 participants (being the model with the second largest evidence for 1 participant; see Fig. 3b). Having established the model with the highest evidence, quantitative changes in coupling were computed for each participant using a mixture of the run-specific changes as specified above (i.e. *adaptation* and *consolidation*) weighted by the appropriate run specific (bilinear) parameter estimates. These estimates (see Fig. 4) provided a quantitative picture of the changes in coupling and its consistency over subjects. Reflecting the characteristics of the winning consolidation model effective connectivity changes were largest between the first and second rest run. They were smaller but consistent for the two rest runs on the second day of scanning, i.e. during rest runs three and four. The same pattern was observed for both directions, i.e. from hippocampus to striatum and vice versa.

## Discussion

We investigated the neural correlates of the rapid perceptual learning phase in a standard visual paradigm and the relationship between learning related changes and spontaneous fluctuations in resting state activity before and after that learning. We showed that a random dot coherence task can be learned by naïve participants within one training session. The task activated primarily visual and parietal brain areas. Significant learning related changes in neural responses were observed in the hippocampus. Furthermore, learning of the task had consequences for resting state connectivity: the hippocampal region changed its coupling with the striatum in a pattern that could be best explained in terms of consolidation. More precisely, a psychophysiological interaction analysis identified learning dependent changes in coupling with the hippocampus that were greater than equivalent changes on the second day without learning. Dynamic causal modeling of the directed interactions between the hippocampal and the striatal region showed that both forward and backward connections expressed learning dependent effects that persisted on the second day. This even allowed non-specific adaptation between paired runs on the two days of data acquisition.

While it is well known that performance on sensory tasks improves with practice, the time course of learning related changes is less established. Unlike ours, many studies do not investigate the early phase of learning, which is usually overlooked due to a familiarization period. This is particularly true for functional MRI studies. While some studies use difficult tasks with training over several days, weeks or even months (Blakemore and Campbell, 1969; Kahnt et al., 2011), rapid learning effects in a number of visual learning tasks have been reported after as few as 200 trials (Fiorentini and Berardi, 1981). Learning of a random dot coherence task, as used in this study, can occur after just 300 trials (Vaina et al., 1995). Using the same 2-alternative-forced-choice paradigm, participants improved their performance in a single session from scoring close to chance to almost perfect. In a follow-up fMRI study Vaina et al. (1998) showed an increase in the activation in V5/MT and a decreased activation of the cerebellum, when comparing neuronal responses during the first task session with responses during the final session. However, the authors did not use any participant-specific performance measurement, whereas here we specifically identified participant-specific learning-related changes over time.

In line with several previous studies, our motion learning task activated visual areas involving V5/MT (Newsome and Salzman, 1993; Rees et al., 2000). The necessary role of the region for motion perception has been established in macaque monkeys and in human patients (Baker et al., 1991; Cowey and Marcar, 1992; Vaina et al., 2001), as well as in healthy humans using transcranial magnetic stimulation (TMS) (Tadin et al., 2011; Walsh et al., 1999). However, we did not find learning related changes (at the relatively conservative statistical threshold employed here) in any visual brain area. This might be due to the fact that our group of learners comprised only 11 participants. Thus, a potentially small effect in visual areas may not have been observed due to a lack of power. Importantly, our main interest here was not the specificity of the learning related effects, but the potential changes in connectivity during rest. For example, such changes are seen in a fronto-parietal network after participants learn a difficult shape identification task (Lewis et al., 2009).

Our finding that early learning dependent effects were seen in the hippocampus supports the idea that sensory learning extends beyond a bottom-up process that is restricted to earlier sensory areas related to the representation of sensory stimuli. Together with previous findings, our results suggest that different sensory learning tasks have different neural correlates in higher level brain areas. In line with several recent studies using electrophysiological and neuroimaging methods, our results are consistent with a role of non-sensory areas in visual decisions and learning (Kahnt et al., 2011; Law and Gold, 2009). Specifically, the role of the hippocampus as the classical area for explicit – or declarative – memory and spatial orientation has been challenged. For example, the MTL (including the hippocampus) is involved in tasks during which participants are not consciously aware of learned contingencies (Rose et al., 2011). Also, several hippocampal and parahippocampal regions including the subiculum change their activity in response to temporal regularities; demonstrating a role for human MTL in statistical learning and providing insight into the formation and evolution of memory representations (Schapiro et al., 2012).

The “classical” distinction between implicit and explicit learning is not straightforward for the motion tasks we used. Implicit learning refers to the incidental learning of complex information; i.e., without awareness of what has been learned (Sun et al., 2008). However, this definition is not uncontroversial (Frensch and Runger, 2003). Typically, three different stimuli structures are used to investigate implicit learning: patterns, sequences and functions (Forkstam and Petersson, 2005). In comparison, explicit learning has been characterized as a process similar to conscious problem-solving used for the control of task variables (Mathews et al., 1989), which gives rise to concrete and conscious knowledge about regularities in the environment (Reber, 1989). It is likely that the early learning phase of our task involved both types of learning. Indeed, the mechanism of any hippocampus-related learning processes does not appear to be sufficiently described by the established dichotomy between explicit/implicit learning. On the one hand, hippocampal activity is associated with perceptual forms of associative learning (Fortin et al., 2002; Van Opstal et al., 2008); on the other, hippocampal involvement is seen for implicit higher-order sequence information (Lieberman et al., 2004), including visual sequence learning (Turk-Browne et al., 2010) and transitive inference tasks (Van Opstal et al., 2008). Furthermore, theoretical and empirical work has characterized the hippocampus as a fast learning system (Colgin et al., 2008; Schendan et al., 2003). We exposed our participants to only one learning session. The observed learning is thus classified as fast, compared to slow and usually small additional improvements over days, weeks or months.

While the traditional view of the role of the hippocampus has linked it to explicit/declarative learning (Neves et al., 2008; Penfield and Milner, 1958; Winocur, 1985) the striatum has been associated with implicit/non-declarative learning (Reiss et al., 2005; Wilkinson and Jahanshahi, 2007). However, as discussed, the classical dichotomy may no longer be tenable for the hippocampus, and may be obsolete for the striatum as well: first, our finding that the connectivity between the hippocampus and the striatum changes in response to learning during rest is in line with earlier findings suggesting that both the hippocampus and the striatum show a dynamic interaction during various types of learning (see Packard and Knowlton, 2002; Poldrack and Packard, 2003 for reviews). Moreover, several neuroimaging studies have examined the role of the hippocampus and the striatum during sequence learning using fMRI (Gheysen et al., 2011; Rose et al., 2011). These results highlight the importance of the MTL system and its connections with the striatum for perceptual learning, independent of its nature; i.e. implicit or explicit. Our finding that connectivity between the hippocampus and the striatum changes is particularly interesting with regard to their role in reinforcement learning. Reinforcement learning describes learning by trial and error to act in a way that maximizes reward (Sutton and Barto, 1998). Previously, several studies have investigated the theoretical and empirical relation between perceptual learning and reinforcement signals (Seitz and Watanabe, 2005; Smith et al., 2009). They showed that reinforcement learning can account for the learning during performance of a visual decision task (Law and Gold, 2009) driven by numerous cortical areas including the striatum (Schultz, 2007).

All these findings – including our own results – indicate that some learning related changes, and in particular early ones, involve non-sensory areas. These might involve an enhanced readout of sensory information as a result of behaviorally improved performance. In other words, fast learning may arise from changes in the interpretation of the respective sensory representation rather than changes in the sensory representation itself. More than that, the distinction between explicit and implicit learning systems seems to become more and more outdated (Rose et al., 2011).

From a methodological perspective, we present a practical example of the use of stochastic DCM for the analysis of fMRI resting state data. Li et al. (2011) established the validity of this method and its ability to model endogenous fluctuations in hidden neuronal states, thereby providing a new perspective on how regionally specific signals in fMRI are generated. Commonly used methods to investigate changes in connectivity are often based on correlations, thereby addressing changes in so-called functional connectivity. However, functional connectivity does not support any conclusions about directionality, whereas DCM allows one to model (context dependent changes in) directed and possibly reciprocal connections between brain areas. In addition to deterministic, i.e. “classical” DCM, the newer stochastic DCM accommodates random fluctuations in hidden neuronal and physiological states. This approach may provide a more plausible perspective on how regionally specific signals in fMRI are generated.

In conclusion, we provide empirical evidence to show that the coupling of spontaneous fluctuations of a brain region engaged in early learning of a sensory task is changed during rest and that these changes persist for at least 24 h. Previously, it has been shown that task performance and/or learning leads to changes in the coupling between brain regions (Seitz et al., 2005; Stevens et al., 2010). Furthermore, performance in a novel perceptual task has been associated with the individual variability in functional connectivity during rest (Baldassarre et al., 2012). Here, we used recent advances in dynamic causal modeling to examine directed changes in brain connectivity in learning-related areas immediately and one day after learning. Our key finding – that the coupling between a hippocampal and a striatal region are best explained by a consolidation model – provides further evidence for the idea that spontaneous fluctuations are continuously updated and modified by experience dependent plasticity. More generally, our findings support the view that the adult brain remains plastic throughout the life-span (May, 2011).

## Figures and Tables

**Table 1:** Main effect of the motion learning task compared to baseline.

	MNI coordinates			t-value	P-value
x	y	z
BA 18 R	− 24	− 94	13	15.43	0.001
BA18 L	15	− 91	− 2	14.48	0.001
Fusiform gyrus R	36	29	− 2	11.35	0.011
Inferior parietal cortex L	− 30	− 46	49	10.58	0.021
Inferior occipital cortex L/MT	− 45	− 67	− 2	10.17	0.030
BA 17/cuneus R	12	− 94	13	10.10	0.032
Inferior orbital frontal cortex L	− 42	20	− 2	9.96	0.036
Medial occipital cortex L	− 42	− 76	7	9.85	0.040
Inferior parietal cortex L	− 27	− 43	40	9.78	0.043

Voxel-level statistics are reported at p < 0.05, FWE corrected. BA = Brodmann area, L = left hemisphere, R = right hemisphere.

**Table 2:** Main effect of the motion learning task compared to the static control task.

	MNI coordinates			t-value	P-value
x	y	z
Inferior parietal cortex R	36	− 37	34	11.81	0.007^a^
Insula R	33	29	1	10.12	0.031^a^
BA 18/19 L	− 24	− 79	13	9.31	P < 0.0001^b^
Inferior parietal cortex L	− 36	− 37	40	9.31	P < 0.0001^b^
Medial cingulate cortex R	9	17	47	8.14	P < 0.0001^b^
Insula L	− 36	20	− 5	7.61	P < 0.0001^b^
Precentral sulcus R	27	− 7	55	6.91	P < 0.0001^b^
Medial frontal cortex R	45	35	31	6.78	P < 0.0001^b^
Inferior frontal gyrus L	− 57	14	28	5.86	P < 0.0001^b^
Caudate nucleus R	15	− 4	19	5.13	P < 0.0001^b^

Voxel-level statistics are reported at ^a^p < 0.05, FWE corrected or ^b^p < 0.0001, unc. BA = Brodmann area, L = left hemisphere, R = right hemisphere.
